# Application of Cupping-Glasses to Abscesses

**Published:** 1830-10-01

**Authors:** 


### LVII.

### Application of the Cupping-glass to the emptying of Abscesses, or other Cavities.

The cupping-glass may be very usefully applied to the above purposes. Our readers are aware that many methods of evacuating large abscesses have been recommended by eminent surgeons. Mr. Abernethy makes a small puncture, carefully squeezes out the pus, and uses every precaution to close the opening. Mr. Brodie is rather disposed to make a larger puncture, and, without any squeezing or kneading whatever, to allow the matter to drain into a fomentation-cloth, taking care to keep the patient in a state of perfect rest. Where many and opposite measures are adopted by men of talent and observation, the result of none is uniformly successful, and we have seen bad consequences ensue after both the methods to which we have alluded. The fact is this: in many cases of psoas or other abscess, the cellular membrane is very extensively destroyed, the muscles perhaps are eaten away, the bones carious, the constitution bad. Now when such is the mischief, bad consequences *must* follow any plan we can devise, or occur if no operation is attempted; the patient being rather destroyed by the disease than by any operative injury inflicted. But still there are milder cases in which the issue is not necessarily fatal, and in which the judicious evacuation of the abscess will effect a cure. It is certain that if such abscesses be opened in an improper and unskilful manner, if there be much squeezing and kneading and probing, the parietes of the cyst inflame, its contents become putrid, sulphuretted hydrogen gas is locked up, and a train of the most characteristic typhoid symptoms are the consequence. Now we have seen Mr. Brodie employ the cupping-glass with much advantage in the emptying of such abscesses. The pressure made by the atmosphere is equable and regular, though strong, and there is less danger of air being admitted through the wound than in the ordinary mode of proceeding. The abscess being freely punctured with a lancet, Mr. Brodie immediately puts on a succession of glasses till the matter ceases, or nearly ceases to escape. He then applies a poultice and keeps the patient in the horizontal posture and in bed. About a month ago we saw a little girl with an abscess on the buttock, apparently communicating with the hip-joint, treated in this manner, and not an unfavourable symptom supervened. We do not pretend that bad consequences will never follow this, as they will any other operation in these cases, but they rarely do so, and the plan is in general unattended with inconvenience. The cupping-glass is equally applicable to the evacuation of other large cavities as to abscesses. Mr. Brodie, for instance, employed it in a case of hydatids on the convex surface of the liver, after puncturing the prominent tumour in the hypochondrium. Perhaps these hints may be of service, and we are sure Mr. Brodie will be pleased to see some of the results of his extensive experience and keen observation, made available and useful to his less fortunate brethren.

